# Deep brain stimulation modulates directional limbic connectivity in major depressive disorder

**DOI:** 10.1017/S0033291725100767

**Published:** 2025-08-08

**Authors:** Egill A. Fridgeirsson, Isidoor Bergfeld, Bart P. de Kwaasteniet, Judy Luigjes, Jan van Laarhoven, Peter Notten, Guus Beute, Pepijn van den Munckhof, Rick Schuurman, Damiaan Denys, Guido van Wingen

**Affiliations:** 1 Department of Psychiatry, University of Amsterdam, Amsterdam, The Netherlands; 2 Amsterdam Neuroscience, Amsterdam, The Netherlands; 3 Department of Radiology and Nuclear Medicine, Isala Hospital, Zwolle, The Netherlands; 4 Department of Psychiatry, ETZ, Tilburg, The Netherlands; 5 Department of Neurosurgery, ETZ, Tilburg, The Netherlands; 6 Department of Neurosurgery, University of Amsterdam, Amsterdam, The Netherlands

**Keywords:** deep brain stimulation, dynamic causal models, major depressive disorder, resting state functional connectivity, effective connectivity, limbic network

## Abstract

**Background:**

Deep brain stimulation (DBS) is being investigated as a treatment for patients with refractory major depressive disorder (MDD). However, little is known about how DBS exerts its antidepressive effects. Here, we investigated whether ventral anterior limb of the internal capsule stimulation modulates a limbic network centered around the amygdala in patients with treatment-resistant MDD.

**Methods:**

Nine patients underwent resting state functional magnetic resonance scans before DBS surgery and after 1 year of treatment. In addition, they were scanned twice within 2 weeks during the subsequent double-blind cross-over phase with active and sham treatment. Twelve matched controls underwent scans at the same time intervals to account for test–retest effects. The imaging data were investigated with functional connectivity (FC) analysis and dynamic causal modelling.

**Results:**

Results showed that 1 year of DBS treatment was associated with increased FC of the left amygdala with precentral cortex and left insula, along with decreased bilateral connectivity between nucleus accumbens and ventromedial prefrontal cortex. No changes in FC were observed during the cross-over phase. Effective connectivity analyses using dynamic causal models revealed widespread amygdala-centric changes between presurgery and 1 year follow-up, while the cross-over phase was associated with insula-centric changes between active and sham stimulation.

**Conclusions:**

These results suggest that ventral anterior limb of the internal capsule DBS results in complex rebalancing of the limbic network involved in emotion, reward, and interoceptive processing.

## Introduction

Major depressive disorder (MDD) is a debilitating psychiatric disorder that is one of the leading causes of chronic disease burden in the world (Ferrari et al., 2013). Standard treatments for patients with MDD include pharmacotherapy, psychotherapy, and electroconvulsive therapy. An investigational treatment option for patients who do not respond to any of these treatments is deep brain stimulation (DBS), which has shown efficacy of around 40% (Bergfeld et al., 2016; Bergfeld & Figee, 2020). In DBS, electrodes are inserted deep into certain brain regions, which can then be stimulated with electrical current. The first brain target for DBS in depression was the white matter of the subcallosal cingulate (Mayberg et al., 2005). Subsequently, different targets have been explored such as the ventral capsule/ventral striatum (Dougherty et al., 2015; Malone et al., 2009), the nucleus accumbens (NAc) (Schlaepfer et al., 2008), the superolateral branch of the medial forebrain bundle (Schlaepfer, Bewernick, Kayser, Mädler, & Coenen, 2013), and the ventral anterior limb of the internal capsule (vALIC) (Bergfeld et al., 2016; van der Wal et al., 2020).

The mechanism by which DBS exerts its antidepressant effects is largely unknown. Mood disorders have been consistently associated with increased activity in the amygdala and insula and decreased activity of the prefrontal cortex and Nac (Simon, Adler, Kaufmann, & Kathmann, 2014; Taylor & Whalen, 2015; Via et al., 2014; Yang et al., 2022). We recently found that long-term vALIC DBS in MDD normalizes amygdala responsivity to emotionally salient stimuli (Runia et al., 2023). In addition, active compared to sham stimulation increased amygdala connectivity with sensorimotor and cingulate cortices. Other studies investigating the neural effects of DBS have used the NAc as a target, which is located just below the vALIC target (Bergfeld & Figee, 2020). Some positron emission tomography studies suggest widespread effects of NAc stimulation on brain metabolism, such as decreases in posterior cingulate, caudate nucleus, thalamus, cerebellum, and dorsomedial prefrontal gyrus and increases in ventral striatum, dorsolateral, and medial prefrontal cortices (Bewernick et al., 2010; Millet et al., 2014; Schlaepfer et al., 2008). However, it remains unknown how DBS affects the interaction between brain networks that are thought to be at the core of depression. We previously investigated how vALIC DBS affects resting state functional connectivity (FC) between the amygdala, the insula, the Nac, and the ventromedial prefrontal cortex (vmPFC) in obsessive compulsive disorder (OCD) (Fridgeirsson et al., 2020), which showed how improvements in mood with DBS were associated with reduced amygdala–insula FC and that DBS decreased directional impact of vmPFC on amygdala and amygdala on the insula. To investigate whether vALIC DBS has comparable effects in MDD, we investigated the same network.

We used two methods to explore this network using resting-state functional magnetic resonance imaging (fMRI). Following our previous studies in OCD, we used seed-based FC analyses using the amygdala and NAc as seeds (Figee et al., 2013; Fridgeirsson et al., 2020). Then, we used an effective connectivity analysis based on spectral dynamic causal modelling (K. J. Friston, Kahan, Biswal, & Razi, 2014). This type of analysis allows us to estimate directional connectivity and excitation/inhibition balance of regions. We tested whether there are connectivity changes from before the DBS surgery and treatment initiation to after the DBS parameters have been optimized to maximize treatment response. We controlled for test–retest effects by measuring healthy controls around the same time points. We then tested whether there are short-term changes during a randomized, double blind cross-over phase where half of the patients are randomized to DBS on, followed by DBS off, and the other half starts with DBS off, followed by on (Bergfeld et al., 2016).

## Materials and methods

### Participants

Patients were recruited at two hospitals in the Netherlands, Academic Medical Center in Amsterdam and St. Elisabeth Hospital in Tilburg. They had a primary diagnosis of MDD with an illness duration of at least two years. They had to be treatment resistant, defined as an inadequate response to at least two different classes of second-generation antidepressants, one tricyclic antidepressant, one tricyclic antidepressant with lithium augmentation, one monoamine oxidase inhibitor and six or more sessions of bilateral electroconvulsive therapy. Symptom severity was assessed using the clinician-rated Hamilton rating scale for depression (HDRS) (Hamilton, 1960) and Montgomery-Asberg depression rating scale (Montgomery & Asberg, 1979) (MADRS) and the self-rated inventory of depressive symptomology (IDS) (Rush et al., 1986). The minimum HDRS score for inclusion was higher than 17, and a Global Assessment of Function score had to be lower than 46 (American Psychiatric Association, 2013). Two electrodes (Model 3389, Medtronic inc.) with four contact points of 1.5 mm, with 0.5 mm spacing, were implanted through the internal capsules. The deepest contact was in the NAc and the others in the vALIC. Patients then underwent an optimization phase where patients were evaluated biweekly, and the stimulation parameters were adjusted to achieve the optimum response. The optimization phase ended after a 4-week stable response or when a maximum of 52 weeks was reached. The location of the active electrodes after optimization phase can be found in Bergfeld 2016 eFigure 2 and eTable 1, and the stimulation parameters are listed in eTable 3 (Bergfeld et al., 2016). After the optimization, the patients underwent a cross-over phase where they were randomly assigned to have DBS on followed by DBS off or vice versa. As described in Bergfeld et al. (2016), the aim was to have each phase 6 weeks, but it could be terminated early if clinically indicated or requested by the patient. Symptom severity was collected at baseline and the end of each phase. Twenty healthy controls were recruited for the imaging comparisons. The study was approved by the medical ethics board of both hospitals (Netherlands Trial Register: NTR2118). All patients and controls signed an informed consent form.

### Image acquisition

Each patient underwent a scanning session preoperatively and a second scanning session after the optimization phase. Each session included a resting state fMRI scan and an anatomical scan. Thereafter, similar scans were obtained with one to six weeks between the scanning sessions, after the end of each cross-over phase. The controls were scanned twice, with one year between sessions. Further details of the image acquisition can be found in the Supplementary Material.

### Image preprocessing

Image processing was performed as described previously (Fridgeirsson et al., 2020). A detailed description is in the Supplementary Material. The functional data was realigned to the first volume. Then, the structural data was brain-extracted and coregistered with the functional data. Then, the data were normalized to MNI (Montreal Neurological Institute) using the unified segmentation (Ashburner & Friston, 2005) approach in SPM12 (http://www.fil.ion.ucl.ac.uk/spm), which is robust to brain lesions which resemble dropout regions due to electrode artifacts (Crinion et al., 2007). The functional data was then resampled to 2 mm isotropic, smoothed with an 8 mm Gaussian kernel and bandpass filtered between 0.01 and 0.1 Hz.

### Functional connectivity analysis

We performed seed-based FC analyses for the laterobasal (LB) amygdala and NAc as these were the regions where previous FC changes had been found using the same target in OCD (Figee et al., 2013; Fridgeirsson et al., 2020). For the LB amygdala, the analysis followed that of Fridgeirsson et al. (2020). For the Nac, it performed similarly, except that the probability map used was from the subcortical atlas (Pauli, Nili, & Tyszka, 2018). A detailed description of the steps is provided in the Supplementary Material.

Since the dropout region due to the electrode artifacts intersects with the Nac, we removed it from the seed region. We subtracted the postoperative normalized mean fMRI from the preoperative scan, which had no dropout. For all subsequent analyses, the dropout regions were removed from the NAc. To further check the effect of this step, we calculated the volume of the removed region and correlations between timeseries from seed regions in preoperative scans including and excluding the dropout. The average proportion of removed volume from the NAc due to dropout was 22% and 20% for the left and right NAc. The average correlation between the NAc and NAc-without–dropout timeseries was 0.84 and 0.89 for the left and right NAc.

To estimate that the effects of motion have been accounted for, the quality control benchmarks from Parkes, Fulcher, Yücel, and Fornito (2018) were used using the parcellation from Gordon et al. (2016). The proportion of significant correlations between framewise displacement and FC was 0.05. The median correlation was 0.11, and the Spearman correlation between the FC and Euclidean distance was −0.06. Compared to the figures in Parkes et al. (2018), this suggests our preprocessing strategy is accounting well for the effects of motion.

For the pre vs post comparison, statistical maps were entered into a 2 × 2 factorial design in SPM12 with factors Group (patient vs controls) and Time (preoperative vs postoptimization). The 2 × 2 interaction approach was chosen because it allows us to directly test whether the change over time in the patient group is significantly different from that in the control group. This is critical given that our control group exhibits systematic test–retest effects (e.g., due to habituation to the MRI environment) rather than random noise, as supported by prior studies (Peters, Cleare, Papadopoulos, & Fu, 2011; Zhang et al., 2019). The factorial design explicitly models both within- and between-group variability, thereby increasing statistical power and providing a more rigorous assessment of treatment-specific effects over and above common test–retest influences. Voxel-wise statistical tests were corrected for family-wise error at the cluster level (*p* < 0.05) with a threshold of *p* = 0.001 (Eklund, Nichols, & Knutsson, 2016), across the entire brain or within a priori regions of interest (Fridgeirsson et al., 2020). Posthoc tests were performed for significant interactions. The same procedure was used for the cross-over phase but with paired t-tests.

### Effective connectivity analysis

To investigate directionality of the connections for the network, we used spectral dynamic causal modelling (DCM) (K. J. Friston et al., 2014). The same procedure as in Fridgeirsson et al. (2020) was followed to build DCM models with four ROIs (left LB amygdala, left NAc, left insula, and vmPFC) as nodes and bilateral connections between all regions defined, resulting in 16 connections, including each region’s self-connection. Insula ROI was defined as a 10-mm sphere on the peak value of FC results from this study. A detailed description of the procedure from (Fridgeirsson et al., 2020) is in the Supplementary Material.

After individual models were inverted, a second-level parametric empirical Bayes (PEB) model was constructed to model the effect of between-subject effects on the estimated effective connections (K. J. Friston et al., 2016; Zeidman et al., 2019). After estimating the group-level PEB model, we performed a search over nested PEB models by pruning those parameters that did not contribute to the model evidence (K. Friston & Penny, 2011; Rosa, Friston, & Penny, 2012). Bayesian model averaging was then used after the final iteration to determine the strength of connections in the last Occam’s window of 256 models. Free energy of models with vs without each connection was used to compute the posterior probabilities (PPs) for each connection. Connections were included in the results if they had very strong evidence (PP > 0.99). PEB is a multivariate Bayesian GLM in which all model parameters are fit at once, and no multiple comparisons correction is required. For the preoperative vs postoptimization comparison, the modelled effect was the interaction of group (patients vs controls) and time (preoperative vs postoptimization). For the cross-over phase, it was the effect of DBS on vs off.

## Results

In 13 of the 25 patients from our study on DBS in depression (Bergfeld et al., 2016), complete imaging sets at baseline and postoptimization were available. Three patients were excluded due to excessive head motion, and in one patient, dicom files were corrupt, resulting in a sample of nine patients. Twenty healthy controls were recruited, of whom three did not complete two scanning sessions. In one, the dicom files were corrupted, and four were excluded due to excessive head motion during scanning, resulting in a sample of 12 controls. The patients and controls did not differ in age, sex, education level, or head motion during scanning (Table 1). The patients’ head motion in the scanner was not significantly different before and after they were implanted with the DBS electrodes (*p* = 0.23, paired t-test). The optimization phase lasted on average 59 weeks (sd: 17.8) and resulted in a significant reduction in MADRS scores. Comparable changes in HAM-D and IDS scores were observed but did not reach significance. Changes in MADRS and HAM-D were highly correlated during optimization (*r* = 0.91) and cross-over (*r* = 0.99), while MADRS/HAM-D were less correlated to IDS during optimization (*r* = 0.87/0.78) than during cross-over (*r* = 0.97/0.98). For the cross-over phase, turning DBS off resulted in significantly worse clinical scores overall (Table 1). For the cross-over phase in our sample, DBS on lasted on average 28.2 days while DBS off lasted on average 12.4 days.

**Table 1.** Demographics of study sample and clinical scales for preoperative vs postoptimization

Demographics long-term comparison	Patients (*n* = 9)		Controls (*n* = 12)		Difference
	Mean (SD)	Range	Mean (SD)	Range	P-value
Age, y.	52.6 (5.5)	47–62	53.3 (8.1)	31–62	0.83^a^
Sex (*n*, % males).	*n* = 2, 22.2%		*n* = 5, 41.7%		0.096^b^
Education level^c^ Illness duration, y	4.2 (2.0) 16.6 (8.3)	2–7 3–31	3.6 (1.7)	2–7	0.44^a^
Motion (mean framewise displacement), mm.	0.22 (0.098)	0.09–0.45	0.19 (0.049)	0.09–0.30	0.15^a^

Clinical scales long-term comparison (*n* = 9)	Preoperative		Postoptimization		P-value
	Mean (SD)	Range	Mean (SD)	Range	
HAM-D	23.3 (6.6)	16–33	15.3 (9.5)	4–34	0.06^d^
MADRS	34.4 (7.8)	23–44	22.6 (13.3)	5–44	0.016^d^ *
IDS	49.7 (7.8)	36–61	38.0 (16.7)	13–71	0.045^d^

Clinical scales cross-over patients (*n* = 9)	DBS on		DBS off		P-value^d^
	Mean (SD)	Range	Mean (SD)	Range	
HAM-D	14.3 (7.3)	5–28	22.4 (5.1)	14–29	0.009*
MADRS	21.9 (12.4)	6–44	34.3 (7.4)	21–44	0.012*
IDS	32.2 (16.4)	10–59	45.9 (9.5)	32–58	0.003*

*Note*: HAM-D, Hamilton Rating scale for depression; MADRS, Montgomery-Asberg depression rating scale; IDS, inventory for depression symptomology.

a independent sample t-test.

b Chi-square test.

c According to the International Standard Classification of Education (ISCED) 2011.

d Paired t-test.

* *p* < 0.05 corrected for three comparisons.

## Functional connectivity

### Preoperative vs postoptimization

**Figure 1.**
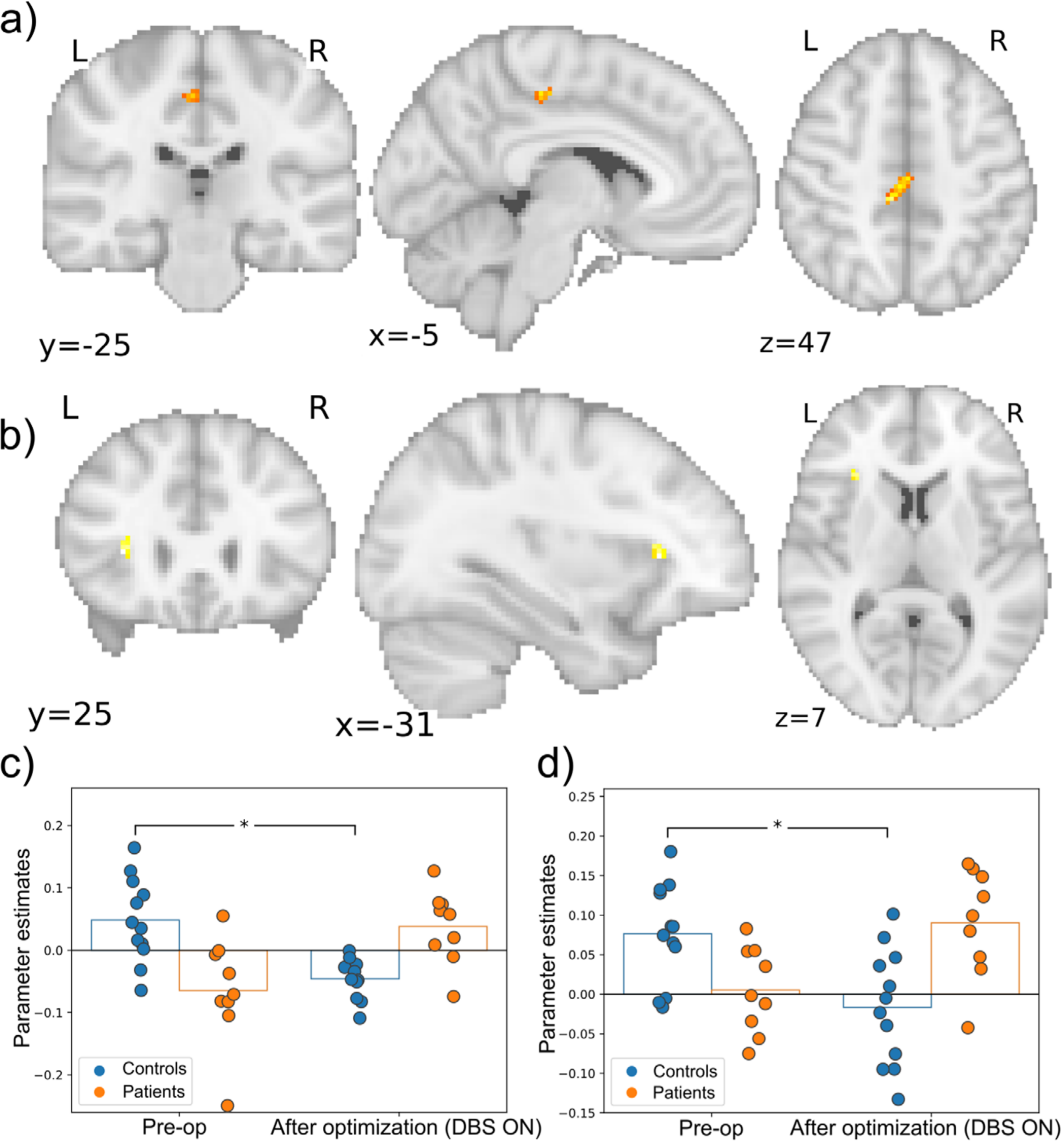
Significant connectivity changes for left LB amygdala seed. (a) Coronal, sagittal, and axial views of the significant cluster in precentral cortex. (b) Coronal, sagittal, and axial views of the significant cluster in the insula. (c) The parameter estimates showing the significance from post hoc testing for the PCC cluster. (d) The parameter estimates showing post hoc significance for the Insula cluster *p < 0.05.

Left LB amygdala connectivity showed a significant interaction between group and time with the left precentral cortex (MNI: (−10, −30, 44), size: 904 mm^3^, *p* = 0.001, FWE-corrected, Figure 1a). The cluster is 23% in the posterior cingulate cortex and 64% in the precentral gyrus (Makris et al., 2006). Posthoc tests revealed a significant decrease of LB amygdala and precentral cortex connectivity in controls over time (*p* = 0.007), whereas it tended to increase (non-significantly) after DBS treatment in patients (Figure 1c). The results in Figure 1c suggest that there was one outlier, though the results remained significant after its removal. The group by time interaction did not reveal any significant clusters for the right LB amygdala as a seed.

In addition, there was a significant interaction between group and time with left LB amygdala connectivity anterior in the left insula (MNI: (−32, 26, 6), size: 136 mm^3^, *p* = 0.027, FWE with small volume (SV) correction; Figure 1b). Posthoc tests with SV correction revealed a significant decrease in controls from the first to the second session (*p* = 0.035). DBS tended to increase the connectivity in patients from preoperative to postoptimization (Figure 1d).

**Figure 2.**
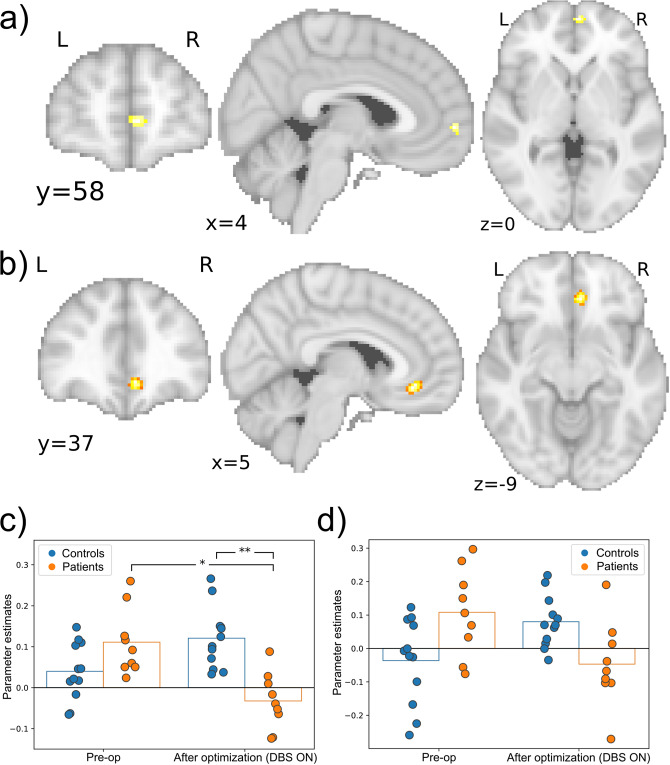
Significant connectivity changes between NAc and vmPFC. (a) coronal, sagittal and axial views of the significant cluster for left NAc seed in the vmPFC. (b) coronal, sagittal and axial views of the significant cluster for right NAc seed in the vmPFC. (c) Parameter estimates for left NAc seed and cluster in vmPFC. (d) Parameter estimates for the right NAc seed and the vmPFC. *p < 0.05, **p < 0.001.

Left NAc connectivity showed a significant interaction between group and time (MNI: (4, 58, 0), size: 176 mm^3^ *p* = 0.013, FWE-correction) with the most anterior part of the right vmPFC (Figure 2a). Connectivity tended to decrease in patients from preoperative to postoptimization, while it increased over time in controls, though these posthoc tests were not significant (Figure 2c).

Right NAc connectivity showed a significant interaction between group and time with the posterior, almost subgenual part of vmPFC (MNI: (6, 36, −10), size: 496 mm^3^, *p* = 0.044, FWE-corrected) (Figure 2b). Posthoc tests revealed a significant decrease in connectivity with this cluster in patients from preoperative to postoptimization (*p* = 0.001). Furthermore, this connectivity was significantly lower in patients than in controls postoptimization (*p* < 0.001; Figure 2d).

### Cross-over phase

For the crossover-phase, FC changes with neither the LB amygdala nor NAc were significant.

## Effective connectivity

### Preoperative vs postoptimization

**Figure 3.**
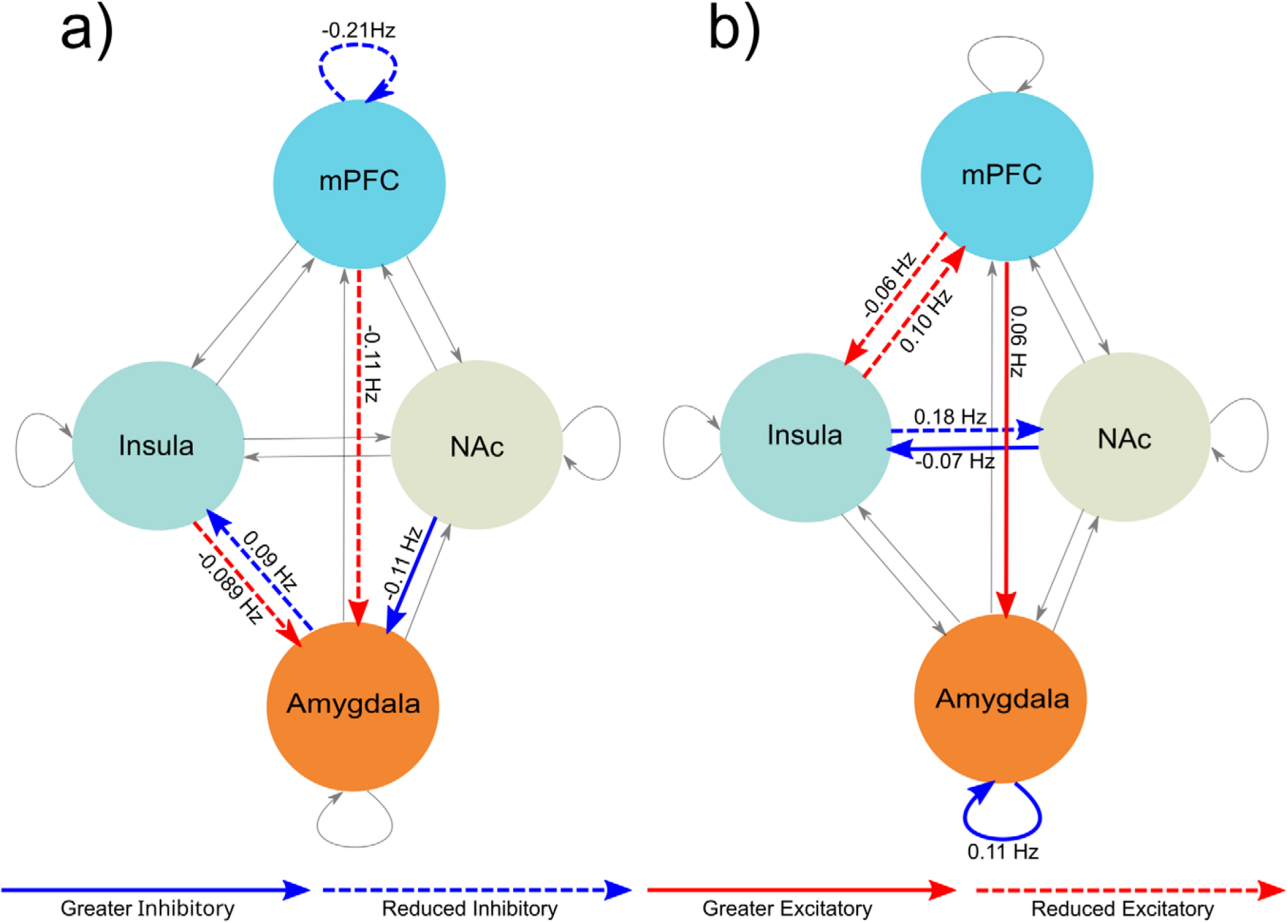
Changes in excitatory and inhibitory connections. (a) After DBS treatment vs pre-surgical network changes compared to changes in controls. (b) Cross-over phase network changes associated with DBS in patients. Grey connections were not significant.

The average explained variance of individual models was 84% with a standard deviation of 4.9%. The results of the PEB model can be seen in Figure 3a and Table 2. The group-time interaction demonstrated changes in network connections in which optimized DBS-treatment was associated with weaker self-inhibition of the vmPFC and weaker excitatory connection from vmPFC to amygdala (−0.11 Hz) in patients compared to controls. After DBS therapy, there was also less inhibitory connection from amygdala to insula (0.09 Hz), with weaker excitatory connection from insula to amygdala (−0.09 Hz), and greater inhibitory connection from NAc to amygdala (−0.11 Hz) in patients compared to controls.

**Table 2.** Parameter estimates for effective connectivity

Connection	Parameter estimates (90% credible interval)	Average connectivity	Direction of changes	Posterior probability
Pre vs post optimization				
NAc → Insula	−0.11 Hz (−0.057 to − 0.16)	Inhibitory	Greater inhibitory	1.0
Insula → Amygdala	−0.089 Hz (−0.035 to − 0.14)	Excitatory	Reduced excitatory	1.0
vmPFC → Amygdala	−0.11 Hz (−0.064 to − 0.16)	Excitatory	Reduced excitatory	1.0
Amygdala → Insula	0.09 Hz (0.024 to 0.156)	Inhibitory	Reduced inhibitory	1.0
vmPFC self-inhibition	−0.21 (−0.29 to − 0.15)	Inhibitory	Reduced inhibitory	1.0
Crossover-phase				
Amygdala self-inhibition	0.11 Hz (0.017 to 0.21)	Inhibitory	Greater inhibitory	1.0
vmPFC → amygdala	0.06 Hz (0.027 to 0.010)	Excitatory	Greater excitatory	1.0
Insula → NAc	0.18 Hz (0.13 to 0.24)	Inhibitory	Reduced inhibitory	1.0
NAc → insula	−0.07 Hz (−0.018 to −0.12)	Inhibitory	Greater inhibitory	1.0
vmPFC → insula	−0.06 Hz (−0.024 to −0.098)	Excitatory	Reduced excitatory	1.0
Insula → vmPFC	0.10 Hz (0.055 to 0.14)	Excitatory	Reduced excitatory	1.0

### Cross-over phase

Average explained variance was 83.7% with a standard deviation of 4.4%. The results for the PEB model for the cross-over are shown in Figure 3b and Table 2. After the cross-over phase with DBS on, there was greater excitatory connection from the vmPFC to the amygdala (0.06 Hz) and with stronger self-inhibition in the amygdala (0.11 Hz). DBS on was also associated with reduced excitatory connection from vmPFC to insula (−0.06 Hz) and from insula to vmPFC (0.10 Hz), as well as with weaker inhibitory connection from insula to NAc (0.18 Hz) and stronger inhibitory connection the NAc to insula (−0.07 Hz).

## Discussion

We investigated the neural mechanism behind the effects of vALIC DBS in MDD in comparison to previous effects found in OCD. DBS treatment in patients increased resting-state connectivity of the left LB amygdala with the precentral cortex and anterior part of the left insula from preoperative to postoptimization in comparison to changes in connectivity in controls. In addition, DBS decreased connectivity between the left and right NAc with the right vmPFC. Effective connectivity modeling using spectral DCM revealed widespread amygdala-centric changes from preoperative to postoptimization in patients compared to controls. However, short-term changes in effective connectivity during the blind cross-over phase primarily revealed reduced insular excitatory connectivity with vmPFC and net insular inhibition from NAc in active compared to sham stimulation.

Once DBS treatment was optimized, FC between left LB amygdala and left insula increased while it decreased between bilateral NAc and vmPFC. In OCD, we observed that vALIC DBS led to comparable changes in NAC-vmPFC connectivity (Figee et al., 2013) but to opposite changes in LB amygdala-insula connectivity (Fridgeirsson et al., 2020). This suggests that vALIC DBS induces generic effects that are independent of the underlying pathophysiology, as well as neural effects that are disorder-specific. Overactive frontostriatal connectivity has been considered a core part of the pathology of OCD (Pauls, Abramovitch, Rauch, & Geller, 2014). For depression, the literature suggests this is not the case. Many studies have reported baseline differences between nonrefractory depressed patients and controls. FC between NAc and vmPFC is lower in patients than controls (Furman, Hamilton, & Gotlib, 2011; Liu et al., 2021; Satterthwaite et al., 2015; B. Zhou et al., 2022). Although differences may be different in refractory MDD, this suggests that the increase in connectivity with vALIC DBS does not normalize these differences in MDD. Moreover, the effect of vALIC DBS on frontostriatal connectivity, now observed in two disorders, could thus be a generic effect of the DBS treatment, which may relate to the implantation of the electrodes close to the NAc.

In OCD, we found decreased LB amygdala-insula connectivity with DBS treatment, which was associated with mood and anxiety changes (Fridgeirsson et al., 2020). Here, we found the opposite effect in MDD, where DBS increased connectivity between these regions. In nonrefractory depression, differences in amygdala connectivity between patients and controls have suggested reduced connectivity in patients (Ramasubbu et al., 2014; Tang et al., 2018). Considering these opposite effects of DBS in the same target across the two disorders, the effects of DBS on this connectivity seem to be dependent on the underlying pathophysiology. It should also be pointed out that the effect in insular connectivity was measured in the most anterior part of the insular cortex in MDD, whereas in OCD, these effects were seen in the posterior area of the insula, suggesting a differential functional organization within the insula. Further, we found increases in LB amygdala – medial precentral gyrus connectivity with DBS, but no such changes were detected in the previous OCD study.

When considering these differences in results between OCD and MDD, we need to keep in mind that the results were obtained using almost opposite study designs. In the current study in MDD, we investigated longer term changes, from preoperative to postoptimization, which can take up to a year. In contrast, in our previous study on OCD, we investigated the effects of turning the DBS device off for up to a week to ten days after one year of active stimulation. Interestingly, we did not observe any significant changes in LB amygdala or NAc connectivity during the cross-over phase in the current study, indicating that these effects in MDD are restricted to the long-term effects of DBS. Thus, while the effects of vALIC DBS on LB amygdala and NAc connectivity appear either independent (NAc) or dependent (LB amygdala) of the underlying pathophysiology, it may be related to short-term effects in OCD and long-term effects in MDD.

The regions implicated with the treatment effect of vALIC DBS in MDD have various roles in the hypothesized mechanism of MDD. The vmPFC is a part of the default mode network, which is associated with increased rumination in the pathophysiology of MDD (Sheline et al., 2009). The NAc is a part of the reward network. Decreased FC between those regions has been implicated with anhedonia in MDD (Liu et al., 2021). It is clear that anhedonia did not increase in our patients with DBS, so it is unclear how our findings match the reported mechanism of MDD.

The amygdala is an important node in the affective processing of the limbic network. We find increases in the connectivity between the LB amygdala and insula with DBS. The LB amygdala is the input and processing node of the amygdala (Roy et al., 2009). The insula has an important role in interoception and the perception of internal feelings (Craig, 2009). In MDD, baseline differences show decreased FC between the amygdala and insula in patients compared to controls (Ramasubbu et al., 2014; Tang et al., 2018). This could indicate impaired top-down emotional regulation. Here, we find increases in this FC, which could indicate some of this impairment is restored. The LB amygdala – sensorimotor cortex connectivity increased during DBS. The cluster indicating increased connectivity with the left LB amygdala was mostly in the medial part of the precentral gyrus (63%) and in the posterior cingulate cortex (23%). While the posterior cingulate cortex is a node of the default mode network (H.-X. Zhou et al., 2020), it is less clear what the role of a motor region such as the precentral gyrus is in depression. However, data-driven resting-state studies show that this is one of the most affected regions in MDD (Gallo et al., 2023; Javaheripour et al., 2021).

For effective connectivity, we found widespread changes both for long-term effects (from preoperative to postoptimization) and during the cross-over phase (Figure 3). The long-term changes are amygdala-centric, while the short-term is more insula-centric. In OCD, we previously found greater excitatory impact of the vmPFC on amygdala and of amygdala on insula with DBS on (Fridgeirsson et al., 2020). Here in the cross-over phase, we do find the same greater excitatory impact of vmPFC on amygdala with DBS. However, the greater excitatory impact of amygdala on insula was not seen. In fact, we found reduced inhibitory impact of amygdala on insula during the long-term comparison. Thus, as in the FC analysis, there are changes which could be a generic effect of vALIC DBS like the increased excitatory impact of the vmPFC on the amygdala and then there are changes that are disorder specific like the increased excitatory impact of amygdala on insula in OCD and reduced inhibitory impact of amygdala on insula in MDD. Additionally, we find in MDD a long-term reduction in the excitatory impact of the insula on the amygdala, along with an increased inhibitory impact of the NAc on the amygdala with DBS. There is a decrease in self-connection of the vmPFC, indicating it is easier for other regions to influence it. Additional findings for the cross-over phase are decoupling between vmPFC and insula and a shift of inhibitory balance between insula and NAc, NAc inhibits insula more, and insula inhibits NAc less. The self-connection on amygdala is strengthened, making it harder to influence. These additional findings in MDD were not found in OCD and thus appear to be disorder-specific.

Studies on effective connectivity in MDD have produced mixed results. Sawada et al. (2022) found decreased impact of the amygdala on the insula in patients with comorbid depression or anxiety, while Kandilarova et al found stronger impact (Kandilarova, Stoyanov, Kostianev, & Specht, 2018). The current study indicates that DBS treatment reduces the influence of these regions on each other, resulting in a decoupling from aversive processing in the amygdala and interoceptive processing in the insula. Another study found baseline differences in the effective connectivity from the vmPFC to amygdala. With controls having a positive connection and patients having a negative one (Almeida et al., 2009). The current study found that this top-down control of the vmPFC on the amygdala was reduced with DBS. None of these studies were performed in treatment-resistant depression. In light of these mixed results, it is not clear how the effective connectivity changes found here can be interpreted in the light of the literature. More studies are needed, especially in the subpopulation of treatment-resistant depression.

There were marked differences between the long-term (preoperative vs postoptimization) and the short-term (crossover-phase) results. For FC, detected changes were for the long-term comparison while for effective connectivity, there were both long- and short-term changes. The long-term changes were amygdala-centric, while the short-term changes were insula-centric. This could indicate that during the optimisation phase, rebalancing of connectivity between the nodes occurs. When entering the cross-over phase, this balance is perturbed in a way that is different from the preoperative baseline. These short-term changes are not detected by FC, only with spectral DCM. While FC measures covariance at zero lag using the blood oxygen level-dependent response, spectral DCM fits the whole cross-covariance and cross-spectrum of the time series, including a model for how the neural activity results in hemodynamic changes. This indicates spectral DCM is a more general connectivity metric that takes more information into account than FC, and our results indicate it can be more sensitive to network changes due to an intervention such as DBS.

The strengths of the study are that we for the first time report the effects of vALIC DBS for depression on resting-state connectivity in both the short and long term. We further limit ourselves to exploring regions that have been implicated using DBS of the same target in OCD (Figee et al., 2013; Fridgeirsson et al., 2020). For the long-term comparison, a particular strength is the inclusion of a control group that allows us to account for repeated testing. MRI testing increases stress, particularly for the first scan in a series, which can have widespread changes on the default function of the brain at rest (Peters et al., 2011; Zhang et al., 2019). In our study, the significant reduction in connectivity between the amygdala and precentral cortex observed in the control group appears to reflect a habituation process, characterised by a decrease in stress-related neural activation with repeated exposure to the scanning environment, rather than an artifact of random noise. By including a control group scanned at the same intervals as the patient group, we were able to account for these test–retest effects and thus more confidently attribute group differences to the stimulation. The resting state acquisition times of 360 seconds at our low sample size have been shown to attain good reliability for DCM (Ma et al., 2024). There are some limitations to our study as well. First, we have a small sample, which unfortunately cannot be prevented, given the small number of MDD patients treated with DBS electrodes. However, to our knowledge, this is the largest sample using functional neuroimaging in DBS for MDD. This study was part of a randomized controlled clinical trial, which was determined to have enough power to detect clinical effects of vALIC DBS in MDD (Bergfeld et al., 2016). The imaging outcomes were secondary outcomes, and unfortunately, complete fMRI data were missing for more than half of the patients. The small sample did not allow for investigating heterogeneity or effects of other factors such as medication use as well as limiting us to detect large effects. Second, because of computational reasons, we cannot include all nodes of interest in our effective connectivity analysis. This could mean that some of the changes we report are caused or mediated by unmodelled nodes. Further, due to the low sample size, we could not analyze the impact of different stimulation parameters on the results.

In conclusion, we found that vALIC DBS treatment in MDD patients modulates resting-state connectivity in a limbic network. Some of these changes appear to be a generic effect of vALIC DBS, while others are disorder-specific. Using effective connectivity, we found long-term changes centered around the amygdala and shorter-term changes which are more insula-centric. This is an indication of complex rebalancing of interceptive and emotional processing changes induced by DBS treatment, which can inform future research to improve DBS treatment for MDD.

## Supporting information

Fridgeirsson et al. supplementary material Fridgeirsson et al. supplementary material

## Data Availability

The subject-level data from this study cannot be shared due to privacy regulations. Derived aggregated data that support the findings of this study are available from the corresponding author on reasonable request.

## Supplementary material

The supplementary material for this article can be found at http://doi.org/10.1017/S0033291725100767.
