# Supplementary material for: Deep brain stimulation modulates directional limbic connectivity in major depressive disorder
Source: Psychol Med. 2025 Aug 8;55:e231. doi: 10.1017/S0033291725100767 (PMC12360689; doi:10.1017/S0033291725100767)
Supplement: Fridgeirsson et al. supplementary material [file S0033291725100767sup001.docx]

**Image Acquisition**

Images were acquired using a 1.5T Siemens MAGNETOM Avanto scanner. A transmit receive head coil was used to minimize exposure of DBS electrodes to the pulsed radiofrequency field. The DBS was turned off a few minutes prior to scanning. The head of the patient was held in place with padding and straps. Specific absorption rate was limited to 0.1 W kg^-1^. Structural images were acquired with 1x1x1 mm resolution using a 3D sagittal MPRAGE with repetition time (TR) of 1.9 s, echo time (TE) of 3.08 ms, flip angle of 15° and inversion time (TI) of 1.1 s. Functional MRI data were acquired with two-dimensional echo-planar imaging with TR = 2000 ms, TE = 30 ms and a flip angle = 90°. Each scan consisted of 25 transversal slices of 4 mm with voxel size of 3.6x3.6 mm and slice gap of 0.4 mm. The first ten volumes were discarded to allow for magnetization stabilization and the subsequent 180 volumes were analyzed. Total scanning time was 360 seconds resulting in 180 3d volumes.

**Analysis pipeline**

**Preprocessing**

Preprocessing was carried out using Nipype, a pipeline tool for neuroimaging data processing (Gorgolewski et al., 2011) and SPM12 (<http://www.fil.ion.ucl.ac.uk/spm>). The functional data were realigned to correct for motion using rigid body realignment to the first functional image. Subjects exhibiting more than 2.5 mm movement in any direction were excluded. After motion correction the brain extracted structural MRI were coregistered to functional space. The structural data were normalized to MNI152 (Montreal Neurological Institute) space using SPM’s unified segmentation (Ashburner & Friston, 2005), which is robust to brain lesions to accommodate the signal dropout due to the DBS system (Crinion et al., 2007). The resulting non-linear warps were then used to normalize the functional data, which were resampled into 2-mm isotropic voxels and subsequently smoothed using an 8-mm Gaussian kernel and bandpass filtered from 0.01 to 0.1 Hz.

**Functional Connectivity**

Data from regions of interest were extracted using SPM’s Anatomy toolbox (Eickhoff et al., 2005). For each amygdala, the centromedial and laterobasal subregions were extracted. The Nucleus Accumbens (NA) was extracted using the probability atlas from Pauli et al (Pauli, Nili, & Tyszka, 2018). The mean signal in the region of interest was computed from the unsmoothed but bandpass filtered functional data. For the subregions the mean signal was weighted with the probability at each voxel as was done in (Roy et al., 2009). This gives stronger weight to those voxels more probable to belong to each subregion. Noise correction followed the procedure described by Muschelli *et al.* (Muschelli et al., 2014). Principal components were extracted from the CSF and white matter signals. The CSF tissue mask from SPM’s unified segmentation was confined to the ventricles using the ALVIN mask (Kempton et al., 2011) and including only voxels with a 99% probability or higher as being CSF. The white matter mask was confined to a 99% probability or higher and eroded to minimize the risk of capturing signals from the nearby grey matter regions. For the signal at each voxel the voxel mean was removed and the results divided by the voxel standard deviation. Singular value decomposition was then used to generate principal components. The components accounting for the 50% of variance from each tissue class were included in the nuisance regression as covariates along with the six motion parameters and derivatives (computed using backward differences). The motion parameters were bandpass filtered to prevent inadvertent reintroduction of nuisance-related variation into frequencies previously suppressed by bandpass filtering (Hallquist, Hwang, & Luna, 2013). For amygdala analysis, the other subregion from the same hemisphere was included in the nuisance regression. After nuisance covariates were regressed out, the region of interest signal was correlated with the remaining residuals from the whole brain. This provided a map of correlation coefficients that were then transformed to *z*-scores using Fisher’s transformation.

**Effective connectivity**

First a general linear model was set up in SPM with cosine basis functions from 1/128 Hz to 0.1 Hz as effects of interest and the movement parameters, white matter and CSF signals as nuisance regressors. This way the resulting effects of interest contrast over the basis functions reveals the resting state fluctuations in that frequency range. For model simplicity only regions of interest from the left hemisphere were included. Regions of interest were the amygdala, insula, vmPFC and NAc. The left amygdala was specified based on an anatomical atlas using the SPM anatomy toolbox. The NAc was defined from (Pauli et al., 2018) excluding the signal dropout due to the DBS electrodes. The vmPFC region of interest was specified as a 10-mm sphere centered on (−1, 49, −5) MNI coordinates (Fox et al., 2005). The left insula region of interest was specified as a 10-mm sphere centred on the peak value in the region of interest from the seed-based correlation analysis. When extracting the signal, the spheres were allowed to shift to the nearest local maxima according to the effects of interest contrast defined above, but within the 10-mm sphere. From each region of interest the first principal eigenvariate, corrected for confounds, was used to represent the region of interest.

Ashburner, J., & Friston, K. J. (2005). Unified segmentation. *NeuroImage*, *26*(3), 839–851. doi: 10.1016/j.neuroimage.2005.02.018

Crinion, J., Ashburner, J., Leff, A., Brett, M., Price, C., & Friston, K. (2007). Spatial normalization of lesioned brains: Performance evaluation and impact on fMRI analyses. *NeuroImage*, *37*(3), 866–875. doi: 10.1016/j.neuroimage.2007.04.065

Eickhoff, S. B., Stephan, K. E., Mohlberg, H., Grefkes, C., Fink, G. R., Amunts, K., & Zilles, K. (2005). A new SPM toolbox for combining probabilistic cytoarchitectonic maps and functional imaging data. *NeuroImage*, *25*(4), 1325–1335. doi: 10.1016/j.neuroimage.2004.12.034

Fox, M. D., Snyder, A. Z., Vincent, J. L., Corbetta, M., Van Essen, D. C., & Raichle, M. E. (2005). The human brain is intrinsically organized into dynamic, anticorrelated functional networks. *Proceedings of the National Academy of Sciences of the United States of America*, *102*(27), 9673–9678. doi: 10.1073/pnas.0504136102

Gorgolewski, K., Burns, C. D., Madison, C., Clark, D., Halchenko, Y. O., Waskom, M. L., & Ghosh, S. S. (2011). Nipype: A flexible, lightweight and extensible neuroimaging data processing framework in python. *Frontiers in Neuroinformatics*, *5*(August), 13. doi: 10.3389/fninf.2011.00013

Hallquist, M. N., Hwang, K., & Luna, B. (2013). The nuisance of nuisance regression: Spectral misspecification in a common approach to resting-state fMRI preprocessing reintroduces noise and obscures functional connectivity. *NeuroImage*, *82*, 208–225. doi: 10.1016/j.neuroimage.2013.05.116

Kempton, M. J., Underwood, T. S. A., Brunton, S., Stylios, F., Schmechtig, A., Ettinger, U., … Simmons, A. (2011). A comprehensive testing protocol for MRI neuroanatomical segmentation techniques: Evaluation of a novel lateral ventricle segmentation method. *NeuroImage*, *58*(4), 1051–1059. doi: 10.1016/j.neuroimage.2011.06.080

Muschelli, J., Nebel, M. B., Caffo, B. S., Barber, A. D., Pekar, J. J., & Mostofsky, S. H. (2014). Reduction of motion-related artifacts in resting state fMRI using aCompCor. *NeuroImage*, *96*, 22–35. doi: 10.1016/j.neuroimage.2014.03.028

Pauli, W. M., Nili, A. N., & Tyszka, J. M. (2018). A high-resolution probabilistic *in vivo* atlas of human subcortical brain nuclei. *Scientific Data 2018 5*, *5*, 180063. doi: 10.1038/sdata.2018.63

Roy, A. K., Shehzad, Z., Margulies, D. S., Kelly, A. M. C., Uddin, L. Q., Gotimer, K., … Milham, M. P. (2009). Functional connectivity of the human amygdala using resting state fMRI. *NeuroImage*, *45*(2), 614–626. doi: 10.1016/j.neuroimage.2008.11.030
